# Violences conjugales à Antananarivo (Madagascar): un enjeu de santé publique

**Published:** 2012-02-13

**Authors:** Bénédicte Gastineau, Lucy Gathier

**Affiliations:** 1 Institut de Recherche pour le Développement, Laboratoire Population-Environnement-Développement CEFORP, Immeuble ENEAM 03 BP 1079 Cotonou, Bénin; 2 Responsable Enquête ELVICA Enda – Bureau Océan Indien, Madagascar

**Keywords:** Madagascar, violence conjugale, femme, santépublique

## Abstract

**Introduction:**

La violence conjugale a été étudiée dans beaucoup de pays développés mais peu en Afrique subsaharienne. Madagascar est un pays où ce phénomène est peu documenté.

**Méthodes:**

En 2007, une enquête sur la violence conjugale à Antananarivo (ELVICA) a été menée sur la violence conjugale envers les femmes dans la capitale malgache. ELVICA a interrogé 400 femmes en union, de 15 à 59 ans. Des informations sur les caractéristiques démographiques, socioéconomiques des couples ont été collectées ainsi que sur les actes de violences physiques des hommes sur leurs épouses. L’objectif de cet article est d’identifier les facteurs de risques de la violence conjugale grave, celle qui a des conséquences sur la santé physique des femmes.

**Résultats:**

Trente-cinq pour cent des femmes qui ont déclaré avoir subi au moins une forme de violence physique au cours des 12 mois précédent l’enquête. Presque la moitié (46%) des femmes violentées ont déclaré avoir déjà eu des hématomes, et environ un quart (23%) des plaies avec saignement. Vingt-deux pour cent ont déjà dû consulter un médecin. Parmi les nombreuses variables socioéconomiques et démographiques testées, quelques-unes sont associées positivement au risque de violence conjugale grave: le fait pour une femme d’être en union consensuelle et d’avoir une activité professionnelle. Il y aussi un lien entre la violence subie et l’autonomie des femmes (liberté accordée par le mari de travailler, de circuler, de voir sa famille).

**Conclusion:**

A Madagascar, comme ailleurs, la lutte contre les violences conjugales est un élément majeur de l’amélioration du statut et de la santé des femmes.

## Introduction

La violence est un problème majeur qui affecte plusieurs milliards de femmes: au niveau mondial, au moins un tiers des femmes ont déjà été battues, contraintes à avoir des rapports sexuels ou maltraitées de quelque autre manière, le plus souvent par quelqu’un de leur connaissance, y compris leur mari [1]. La famille et plus particulièrement le couple sont en effet les lieux où les violences exercées sur les femmes sont les plus nombreuses [1]. Injures, propos blessants, humiliations, jalousie excessive, interdiction de sortie, confiscation des revenus, gifles, coups, viols, les formes de violence conjugale sont multiples et elles compromettent fortement la vie sociale, l’insertion économique et politique des femmes. La violence envers les femmes est également une question de santé publique, un facteur majeur de morbidité et de mortalité [2–5] et un obstacle supplémentaire à l’application des droits en matière de santé sexuelle et reproductive [6,7].

Parallèlement à un mouvement mondial de reconnaissance des violences contre les femmes, les enquêtes et recherches visant à mieux évaluer et expliquer le problème se sont multipliées dans les pays du Nord où nous disposons maintenant de statistiques nationales [8]. Dans les pays du Sud, les enquêtes portent souvent sur des échantillons de population bien spécifiques (les jeunes, les femmes enceintes, les femmes porteuses du VIH-Sida, etc.) et permettent rarement des comparaisons internationales ou même continentales. Il faut citer toutefois l’enquête de l’Organisation Mondiale pour la Santé (2005) sur la santé et la violence domestique à l’égard des femmes, qui porte sur 10 pays représentant des contextes culturels divers (le Bangladesh, le Brésil, l’Ethiopie, le Japon, la Namibie, le Pérou, la Tanzanie, le Samoa, la Serbie et Monténégro et la Thaïlande) [9]. Une même enquête a été menée dans des régions (rurales et urbaines) de ces dix pays. Elle confirme que la violence domestique existe partout mais que ce type de violence est nettement plus fort dans certains pays. Dans les zones rurales de l’Ethiopie par exemple, 29% des femmes ont été victimes de violences physiques et 44% de violences sexuelles au cours des 12 mois précédant l’enquête. Dans les villes japonaises concernées par l’enquête OMS, les chiffres sont respectivement de 3% et 1% [9].

Au sein des pays du Sud, l’Afrique sub-saharienne francophone se distingue: les statistiques et les études sur la violence domestique envers les femmes sont encore rares, voire inexistantes dans certains pays. Sur le continent africain, beaucoup des recherches sur la violence se focalisent sur les situations de conflit [10,11] ou sur le lien entre VIH et violence sexuelle [12–14] et donc sur l’Afrique anglophone. L’enquête multipays de l’OMS couvre certes trois pays africains (Ethiopie, Namibie, Tanzanie) mais tous sont anglophones [9]. On sait peu de choses sur la prévalence de ce type de violence dans l’espace francophone. De même, les causes et les facteurs de risque associés à la violence domestique et conjugale sont encore mal connus [15]. Beaucoup de recherches s’accordent sur le fait qu’ils dépendent du contexte social et culturel, des normes qui régissent les relations entre les genres et de l’ensemble des inégalités entre les sexes (accès sexué à la scolarisation, au marché du travail, etc.) [15–18]. Une même caractéristique démographique ou socioéconomique peut alors être ou ne pas être un facteur de risque selon le contexte. Par exemple, l’éducation semble protéger les femmes contre la violence conjugale en Tanzanie : les femmes qui ont fréquenté l’école secondaire sont deux fois moins touchées par la violence conjugale que les femmes peu ou pas scolarisées [19]. A l’inverse, Anderson et al. (2007) montrent l’absence de lien entre le niveau d’éducation et le risque de violence sur 8 pays d’Afrique [17]. De même, la question de l’autonomie financière est complexe: en Chine par exemple, moins la femme contribue aux dépenses du ménage, plus elle est susceptible de subir des violences [4] à l’inverse, en Tanzanie, la violence conjugale est surtout importante dans les ménages où les revenus sont assurés par les femmes [18] et en milieu rural, au Bangladesh, les femmes les plus autonomes vis-à-vis de leur époux sont aussi celles qui sont le plus violentées.

A l’instar des autres pays d’Afrique sub-saharienne, les questions de violence envers les femmes à Madagascar sont peu documentées. Une étude récente montre toutefois que les violences domestiques ont des conséquences importantes sur la santé des femmes. Une analyse des registres d’admission du service des urgences du plus grand hôpital d’Antananarivo (HJRA) permet d’apprécier l’ampleur du phénomène [20]. Du 1er janvier au 30 juin 2006 : 1551 femmes victimes de violence ont été accueillies aux services des urgences du CHU. Plus de la moitié des agressions ont lieu au domicile même de la femme (55%) et seul un quart (27%) des violences se déroulent dans un lieu éloigné du foyer de la victime. Les agresseurs sont souvent des proches. Les conséquences des violences sur la santé des femmes sont importantes: 44% des femmes sont venues à l’hôpital avec des contusions et 55% avec des lésions céphaliques (dont 34% avec des traumatismes crâniens). Des lésions graves (fractures ou traumatismes crâniens graves) ont été diagnostiquées chez 2% des patientes.

Si les registres des structures médicales nous renseignent utilement sur les conséquences graves de la violence conjugale, ils nous disent peu sur la fréquence des violences et le contexte socioculturel dans lequel elles s’exercent. C’est pourquoi une enquête sociodémographique a été menée à Antananarivo capitale de Madagascar, en 2007. L’enquête sur la violence conjugale à Antananarivo (ELVICA) permet de mesurer la prévalence des violences psychologiques, physiques, sexuelles subies par les femmes en union au cours de l’année précédent l’enquête et d’identifier des facteurs de risque.

Après avoir présenté la méthodologie de l’enquête ELVICA, nous donnerons quelques résultats sur la population étudiée et les prévalences de violence conjugale. Ensuite, nous focaliserons notre attention sur la violence conjugale grave, celle qui blesse physiquement les femmes (hématomes, plaies avec saignement, fractures, etc.), pour rechercher quelques-uns des facteurs de risque de ce type de violence. Enfin, nous discuterons nos résultats au regard du contexte socioculturel malgache et d’autres études menées en Afrique avant de conclure.

## Méthodes

L’enquête ELVICA a été initiée et financée par le laboratoire Population-Environnement-Développement (LPED) de l’Institut de Recherche pour le Développement (IRD, Marseille, France) et par le bureau Océan Indien de l’ONG ENDA (Antananarivo, Madagascar). Elle s’est déroulée dans la commune urbaine d’Antananarivo entre le 10 et 25 juillet 2007. Quatre-cents femmes résidentes ont été interrogées. Il s’agit de femmes âgées de 15 à 59 ans, mariées ou en union libre au moment de l’enquête (depuis au moins 3 mois) ou vivant seules mais ayant cohabité avec un conjoint au moins 3 mois au cours des 12 derniers mois. Les 400 femmes ont été sélectionnées à partir d’un échantillon de ménages établi sur les 6 arrondissements de la ville. L’échantillon a été constitué par un tirage aléatoire à partir d’une liste de ménages fourni par les présidents de quartiers. Dans chacun des ménages tirés, nous avons interrogé le chef de ménage si celui-ci était une femme ou son épouse dans le cas contraire sous réserve que la femme remplisse les conditions en termes d’âge et de statut matrimonial. Il faut préciser que la quasi-totalité des ménages à Antananarivo sont mononucléaires, il est exceptionnel que deux femmes mariées cohabitent dans le même logement. L’échantillon a été réalisé sous la responsabilité d’un statisticien de l’Institut National de la Statistique de Madagascar et avec le concours des services municipaux de la ville d’Antananarivo.

Toutes les femmes enquêtées ont préalablement donné leur consentement avant d’être interrogées. Seules deux femmes ont refusé de participer à l’enquête. La collecte de données s’est faite à l’aide d’un questionnaire ne comprenant que des questions fermées et en langue malgache. Considérant que le terme « violence » peut impliquer des interprétations variées et compromettre la qualité des réponses obtenues, nous avons adopté une méthode consistant à ne pas nommer la violence dans les questions posées, ceci afin d’éviter au maximum le risque de sous-notification dans les résultats.

Le questionnaire administré comportait des questions sur la composition du ménage, les caractéristiques démographiques, socioéconomiques et sur l’histoire matrimoniale de la femme et de son conjoint, sur les violences subies durant les 12 mois précédent l’enquête, sur les sujets de conflits entre époux ainsi que sur l’histoire génésique de la femme. Les violences sur lesquelles ont été interrogées les femmes sont uniquement celles subies au cours des 12 mois précédent l’enquête et celles perpétuées par leur conjoint (mari ou concubin). Elles peuvent être classées en trois groupes : les violences psychologiques, les violences sociales et économiques, les violences physiques, les violences sexuelles.

Le travail de collecte de données a été réalisé par une équipe de 9 femmes assistantes sociales formées à la collecte de données. Les récents travaux de recherche sur la violence contre les femmes ont soulevé de nombreuses questions éthiques portant tant sur la sécurité des femmes enquêtées que sur celle des chercheurs et des enquêteurs. Des programmes de recherche ont aussi posé la question du traumatisme qui peut être provoqué par le fait de raconter ou d’écouter des épisodes de violence [19]. Nous avons été particulièrement attentifs à respecter la confidentialité des entretiens, à rassurer les femmes sur l’anonymat des questionnaires et à laisser aux enquêtrices suffisamment de temps libre entre chaque enquête. Les femmes ont toujours été interrogées à un moment où elles étaient seules. Lorsque cela n’était pas possible dans le logement du ménage, l’entretien a pu se faire dans un autre lieu choisi par la femme. Les enquêtrices disposaient d’un questionnaire factice sur « Les conditions de vie des ménages à Antananarivo » qu’elles pouvaient sortir si un individu faisait irruption pendant l’entretien de façon à ce que ce dernier ne sache pas sur quoi la femme était interrogée. De plus, à toutes les femmes déclarant avoir été victimes de violence, les enquêtrices ont proposé un ensemble de documents, de contacts d’associations pour les renseigner sur leur droit et les recours possibles. D’une façon générale, nous nous sommes appuyés sur le guide de recommandations rédigé par l’OMS sur les principes de sécurité et d’éthique à respecter lors de cette enquête [21].

Les questionnaires complétés ont été contrôlés puis saisies dans une base de données. Les analyses des données - tableaux de fréquence, tableaux croisées et régressions logistiques - pour cet article ont été réalisées avec le logiciel SPSS.

## Résultats

### Caractéristiques démographiques et socioéconomiques des femmes

L’âge médian des 400 femmes enquêtées est de 32 ans (Tableau 1). Près des trois quarts des femmes interrogées étaient mariées au moment de l’enquête, 21% vivaient en concubinage et 4% vivaient seules mais avaient été en couple durant l’année précédente. Plus de trois quarts des femmes sont entrée en première union avant l’âge de 25 ans. Les femmes sont peu nombreuses à n’avoir jamais été à l’école et près de trois quarts d’entre elles ont même achevé le cycle de l’école primaire. Quatorze pour cent bénéficient d’un niveau d’instruction élevé : elles sont titulaires du baccalauréat et ont pour certaines poursuivi leurs études à l’université.

**Tabelau 1:** Répartition des femmes selon différentes caractéristiques (%), Antananarivo, 2007 (Effectifs : 400 femmes)

		%
**Tranches d’âge (années)**	15–19	5,8
	20–24	11,0
	25–29	23,8
	30–34	17,8
	35–39	17,3
	40–44	13,3
	45–49	6,5
	50–59	4,8
	Total	100,0
**Situation matrimoniale**	Mariée	74,5
	En union libre	21,0
	Seule	4,5
	Total	100,0
**Age d’entrée en première union**	<16	4,0
	16–19	34,5
	20–24	39,0
	25–29	14,5
	>30	7,8
	NSP	0,3
	Total	100,0
**Niveau d’instruction**^1^	Très faible	26,2
	Faible	34,2
	Moyen	24,7
	Elevé	14,0
	NSP	0,8
	Total	100,0
**Revenu mensuel de la femme (en ariary)**^2^	Aucun	40,0
	< 61000	35,3
	61000–120000	14,0
	> 120000	9,3
	NSP	1,5
	Total	100,0
**Revenu mensuel du couple (en ariary)**	0–79 000	17,0
	80000–125 000	28,8
	126000–399 000	38,0
	400000 et plus	7,3
	NSP	9,0
	Total	100,0
**Nombre de pièces du logement**	1	43,5
	2	25,3
	3	16,3
	4 et plus	15,0
	Total	100,0
**Nombre de pièces du logement par personne**	< 0,25	22,3
	0,25 à 0,5	36,5
	0,5 à 0,9	30,3
	1 et plus	11,0
	Total	100,0

1: Très faible: n’avoir jamais été à l’école ou ne pas avoir achevé le cycle de l’école primaire. Faible : avoir achevé l’école primaire (avec obtention du certificat d’études primaires). Moyen: avoir été en second cycle (collège, lycée) sans obtenir le baccalauréat. Elevé: avoir obtenu le baccalauréat.

2: 61000 ariary mensuel correspond au moment de l’enquête à un dollar par jour. Source: ELVICA, 2007

Les niveaux de revenus des femmes sont faibles. Quarante pour cent ne disposent même d’aucun revenu. Ces femmes se déclarent généralement femme au foyer. Les activités féminines sont majoritairement informelles (petits commerces alimentaires, couturières, etc.), elles ne dégagent que peu de revenus qui de plus sont très irréguliers. Trente-cinq pour cent gagnent moins de 61 000 ariary mensuellement (soit moins de un dollar par jour) et seules 9% dépassent le seuil des 120 000 ariary par mois. Les revenus des femmes et des ménages sont un indicateur de la grande pauvreté et précarité dans lesquelles vivent les couples interrogés. Ceci se traduit par des conditions de vie difficiles : beaucoup de couples vivent dans une seule pièce (43%) et ce même quand la famille comprend plusieurs enfants. Près de 60% des familles disposent au maximum d’une demi-pièce par personne.

### Prévalence de la violence physique

Les résultats de l’enquête ELVICA ont montré une très forte prévalence de la violence conjugale à Antananarivo : 35% des femmes ont eu à subir des violences physiques au cours des 12 mois précédent le passage des enquêtrices (Tableau 2). La violence la plus fréquente est la gifle : 27% des femmes ont été giflées au moins une fois. Seize pour cent ont été empoignées ou bousculées brutalement et 17% ont été frappées. Enfin 8% ont même été frappées avec un objet et 3% avec une arme. Ces violences physiques peuvent avoir des conséquences très graves sur la santé des femmes. Presque la moitié (46%) des femmes violentées physiquement ont déclaré avoir déjà eu des hématomes, environ un quart (23%) des plaies avec saignement, 15% ont eu un membre foulé ou fracturé (Tableau 3). Vingt-deux pour cent ont déjà dû consulter un médecin pour soigner des blessures suite à une violence conjugale et 29% ont été dans l’incapacité d’aller travailler après avoir été frappées par leur mari. Au total, 19% des enquêtées (77 sur 400) ont victimes de ce que nous nommerons des violences conjugales graves, c’est-à-dire violences avec blessures (hématomes, plaies, fractures, brûlures).

**Tableau 2:** Proportion des femmes (%) ayant subi différents types de violence au cours des 12 derniers mois, Antananarivo, 2007 (Effectifs : 400 femmes)

	%
La femme a été giflée	27,2
La femme a été bousculée ou empoignée brutalement	16,2
La femme a été frappée (sans que l’homme n’utilise d’objet)	17,2
La femme a été frappée avec un objet	8,8
La femme a été frappée par une arme	2,7
Femmes ayant subi au moins une de ces violences	35,5

Source: ELVICA, 2007

**Tableu 3:** Parmi les femmes ayant subi de la violence conjugale au cours des 12 mois précédent l’enquête, proportion (%) de celles qui ont été blessées par leur conjoint, Antananarivo, 2007. (Effectifs : 142 femmes)

	%
A eu des hématomes	45,8
A eu des plaies avec saignement	23,2
A eu une fracture ou foulure	14,7
A été brulée	3,5
A consulté un médecin	22,5
A été dans l’incapacité de travailler	28,9

Source: ELVICA, 2007

### Facteurs de risque de la violence conjugale grave

Il s’agit maintenant d’identifier parmi les caractéristiques des femmes et de leur conjoint les facteurs de risques associés à la violence conjugale grave, celles qui a des conséquences sur la santé physique des épouses. Par conséquent, nous avons réalisé des régressions logistiques permettant de calculer l’effet d’un grand nombre de facteurs potentiels sur les risques pour une femme de subir ce type de violence. Notre variable dépendante est définie ainsi : 0=pas de violence physique conjugale ayant entraîné de blessures au cours des 12 mois précédent l’enquête ; 1=un ou plusieurs épisodes de violence conjugale grave (ayant entraîné des blessures) au cours des 12 mois précédent l’enquête. Les coefficients sont exprimés en Odd ratio en référence à la catégorie de référence.

Beaucoup de variables démographiques et socioéconomiques, caractéristiques des femmes, des hommes ou du couples ont été testées (Tableau 4). Toutes ne se sont pas révélées prédictives du risque de violence conjugale grave. Le statut matrimonial, l’activité et le revenu de la femme ainsi que l’activité du conjoint ont un effet significatif sur la violence conjugale. Les femmes en union libre ont un risque d’être victimes de violence grave 2 fois plus important que celles qui sont légalement mariées (Odd ratio 2,065). Les femmes sans conjoint sont nombreuses à avoir déclarées des violences au cours de l’union précédente : on peut supposer que ces dernières sont une des causes récurrentes des dissolutions des unions.

**Tableau 4:** Risque relatif (odd ratio) pour une femme d’avoir été victime de violence grave (violence physique avec blessures) selon différentes caractéristiques sociodémographiques

Caractéristiques socioéconomiques	%	Effectifs	OR	P-value	Intervalles de confiance 95%
**Caractéristiques de la femme**					
**Age**					
Moins de 25 ans	21,2	66	1,337	0,425	0,656–2,725
25–34 ans	21,1	161	1,329	0,310	0,767–2,303
35 ans et plus	16,8	173	Réf.		
**Statut matrimonial**					
Mariée	15,4	298	Réf.		
Célibataire en union libre	27,3	84	2,065	0,003	1,164–3,664
Divorcée, veuve, célibataire vivant seule	44,4	18	4,381	0,013	1,642–1,690
**Nombre d’unions**					
Une union	20,3	69	Réf.		
Deux et plus	19,0	331	0,924	0,810	0,483–1,765
**Niveau instruction**					
Sans instruction ou primaire incomplet	22,7	110	Réf.		
Primaire complet et secondaire incomplet	13,7	137	0,633	0,150	0,333–0,180
Secondaire incomplet et supérieur	15,7	153	0,873	0,663	0,475–1,606
**Nombre d’enfants nés vivants**					
0–1	15,0	87	0,842	0,601	0,443–1603
2–4	17,2	232	Réf.		
5 et plus	19,8	81	0,995	0,995	0,527–1,878
**Activité au moment de l’enquête**					
Commerce (secteur informel)	25,9	85	2,328	0,013	1,1195–4,540
Fonction publique, santé et éducation secteur privé	8,3	24	0,606	0,518	0,133–2,767
Artisanat	23,6	110	2,063	0,025	1,093–3,896
Sans activité	13,0	161	Réf.		
Autres	30,0	20	2,857	0,052	0,989–8,252
**Revenu**					
La femme n’a pas de revenu	22,5	160	Réf.		
La femme a un revenu personnel	14,5	240	1,728	0,045	1,012–2,953
**Niveau de revenu mensuel**					
Aucun revenu	22,5	160	Réf.		
Faible revenu (Inf. à 50 000 Ariary)	26,5	83	2,147	0,023	1,112–4,145
Revenu moyen (50 000 à 100 000 Ariary)	22,9	96	1,770	0,085	0,925–3,388
Revenu élevé (Sup. ou égal à 100000 Ariary)	16,9	59	1,215	0,638	0,540–2,754
					
**Caractéristiques de son conjoint**					
**Age**					
Moins de 35 ans	21,2	146	1,805	0,761	0,641–1,837
35–49 ans	19,9	210	Réf.		
50 ans et plus	10,0	50	0,447	0,110	0,167–1,200
**Niveau instruction**					
Sans instruction ou primaire incomplet	27,4	55	1,929	0,802	0,860–4,329
Primaire complet et secondaire incomplet	16,4	135	Réf.		
Secondaire incomplet et supérieur	15,0	187	0,900	0,111	0,397–2,043
**Activité au moment de l’enquête**					
Commerce (secteur informel)	25,7	111	Réf.		
Fonction publique, santé et éducation secteur privé	5,3	38	0,161	0,016	0,370–0,711
Artisanat	19,9	176	0,694	0,204	0,395–1,219
Sans activité	15,6	41	0,596	0,269	0,238–1,491
Autres	17,1	32	0,536	0,242	0,189–1,525
					
**Caractéristiques du couple**					
**Ecart d’âge entre l’homme et la femme**					
Ecart négatif ou nul	24,4	86	1,793	0,070	0,954–3,370
Ecart entre 1 et 5 ans	21,8	124	1,545	0,143	0,864–2763
Ecart supérieur à 5 ans	15,3	190	Réf.		
**Ecart de niveau de scolarisation entre l’homme et la femme**					
Les deux ont le même niveau	21,1	157	1,073	0,801	0,619–1,859
La femme a un niveau supérieur	14,9	87	0,709	0,341	0,349–1,439
L’homme a un niveau supérieur	19,9	156	Réf.		

*p< 0,05; ** p< 0,01

Les tananariviennes qui ont une activité professionnelle au moment de l’enquête sont significativement plus à risque que celles qui sont sans activité, tout particulièrement si elles ont des emplois précaires : artisanat (Odd ratio 2,063) ou commerce informel (Odd ratio 2,328). Ces deux catégories regroupant 81% des femmes ayant une activité économique. Il n’est pas surprenant alors de constater que les femmes ayant des revenus faibles (inférieurs à 61 000 ariary par mois) ont un risque de connaître des violences physiques avec blessure plus élevé (Odd ratio 2,147) que celles qui n’ont aucun revenu. Il faut noter que le fait d’avoir un revenu élevé ne diminue significativement le risque. Concernant le conjoint, l’âge et le niveau d’éducation ne sont pas statistiquement liés à la violence conjugale que nous étudions. En revanche, le fait d’avoir un conjoint fonctionnaire, ou travaillant dans les secteurs de la santé et de l’éducation (secteurs privé et public) réduit considérablement le risque pour la femme de connaître des violences (Odd ratio 0,161). Les écarts d’âge ou de niveau d’éducation entre la femme et l’homme n’ont pas d’effet significatif.

Dans un second temps, nous avons testé des variables de comportement des hommes (Tableau 5) : lors de l’enquête nous avions demandé aux femmes si au cours des 12 mois précédent l’enquête, leur mari était jaloux (par exemple s’il leur interdisait de parler à d’autres hommes), s’il les empêchait de voir leurs amis, leur famille et enfin si leur mari contrôlait et surveillait leurs déplacements. Cinquante-deux pour cent de l’ensemble des enquêtées ont déclaré que leur mari avait été jaloux, 23% que leurs fréquentations avaient été limitées et 36% que leurs déplacements avaient été contrôlés. Ces atteintes aux libertés des femmes sont fortement liées à la violence physique que nous qualifions de grave (Tableau 5). Les épouses dont le conjoint leur a interdit de parler ou de voir d’autres hommes ont presque 4 fois plus de risque d’être victime de violence (Odd ratio 3,784), celles dont les visites aux amis et à la famille sont restreintes ont un risque multiplié par 3 (odd ratio 3,117) et enfin pour celles qui ne peuvent pas se déplacer librement, le risque est presque 3 fois supérieur (odd ratio 2,864) (Tableau 4).

**Tableau 5:** Risque relatif (odd ratio) pour une femme d’avoir été victime de violence grave (violence physique avec blessures) selon différents indicateurs des relations entre conjoints

Indicateurs de relations entre conjoints	%	Effectif	Odd Ratio	P value	Intervalles de confiance 95%
Le mari est jaloux : il interdit à sa femme de parler à d’autres hommes					
Oui	28,4	190	3,784	0,000	2,137–6,701
Non	9,5	208	Réf.			
Le mari empêche sa femme de voir ses amis et sa famille					
Oui	34,8	92	3,117	0,000	1,829–5,311
Non	14,6	308	Réf.			
Le mari contrôle les déplacements de sa femme					
Oui	29,9	144	2,864	0,000	1,718–4-777
Non	12,9	255	Réf.			

## Discussion

L’enquête sur la violence conjugale confirme tout d’abord que les violences conjugales sont très fréquentes à Antananarivo: 35% des femmes ont subi de la violence physique au cours des 12 derniers mois. Il est donc pertinent de mener des recherches sur les questions de violence envers les femmes en dehors de toute situation de conflit ou d’épidémie du VIH.

La comparaison de ces résultats avec ceux d’autres enquêtes est difficile. Les résultats des enquêtes sur ce sujet sensible sont très dépendants des choix méthodologiques et éthiques préalables [7]. Toutefois, on peut affirmer qu’à Madagascar, comme en Afrique plus généralement ou en Europe, l’espace conjugal est un lieu où les violences envers les femmes sont donc très nombreuses [8,9] alors même que la société malgache a souvent été présentée comme une « société sans violence » [22], tout particulièrement le groupe des merina vivant dans la région d’Antananarivo. Le principe du Fihavanana, règle sociale qui définit le mode de relations entre les individus, impose notamment au garçon de « masquer ses sentiments, (de) maîtriser ses émotions, ses pulsions agressives » {22 : 564}. Le résultat est une cohésion sociale admirable qui donne l’image d’une société malgache calme, polie et non violente [23]. Pourtant, l’apparente cohésion sociale ne perdure que sous conditions de sanctions faites aux personnes qui s’écarteraient du Fihavanana et qui voudraient exister par elles-mêmes ; ces sanctions peuvent être d’une violence extrême [23]. Les relations entre les hommes et les femmes n’échappent pas à cette règle. Les rôles dévolus à chacun des sexes, les normes et valeurs intégrées dès la plus petite enfance par les hommes et les femmes instruisent un système de genre qui met les femmes en position d’infériorité tant dans la sphère publique (marché du travail, pouvoir politique, etc.) que dans la sphère privée [24]. Tout écart à ces normes de genre génère des conflits et des violences entre époux.

Les résultats de l’enquête ELVICA montrent ensuite que la violence conjugale est un problème de santé publique : 19% des femmes ont été blessées par leur conjoint au cours des 12 mois précédent l’enquête. Ces résultats concordent avec d’autres études sur l’Afrique [16]. La violence conjugale a par conséquent des coûts directs (soins de santé par exemple) et indirects : presque un tiers des femmes blessées ont été dans l’incapacité temporaire de travailler. Cette violence réduit les possibilités d’épanouissement personnel de la femme et affecte parfois gravement sa santé physique et mentale.

Enfin, aucun groupe de femmes n’est totalement épargné par la violence conjugale. Cependant nous avons pu observer certains facteurs de risque. Les femmes en union consensuelle sont plus à risque que les femmes légalement mariées. Ceci a été constaté dans d’autres contextes africains [16]. De même, les femmes qui ont une activité économique et un revenu sont plus fréquemment victimes de violence conjugale grave. Les conflits et donc les violences sont plus nombreux dans les couples où l’épouse s’éloigne du modèle, idéal type qui voudrait que l’épouse joue le rôle de la femme au foyer en y élevant ses enfants et en dépendant financièrement de son époux {25 : 184}. Les occasions qui sont données à la femme de rencontrer d’autres hommes, de disposer d’un salaire propre sont autant d’occasion de jalousie, de suspicion d’infidélité de la part des maris et elles peuvent générer de la violence. La question de la jalousie est un élément souvent évoqué dans les enquêtes portant sur les violences entre conjoint [15, 26, 27].

Notre étude comporte néanmoins des limites. Premièrement, comment toutes les enquêtes sur la violence, il est probable que les faits de violence aient été sous-estimés. Les femmes peuvent être réticentes à les déclarer malgré toutes les précautions méthodologiques et logistiques qui ont été mises en ’uvre. Deuxièmement, les facteurs de risque de la violence conjugale sont très nombreux et non pas tous été explorés dans l’enquête ELVICA: nous ne connaissons rien par exemple de l’exposition à la violence pendant l’enfance des enquêtées; la consommation d’alcool des conjoints, variable identifiée comme facteur de risque dans beaucoup d’études [15, 16, 28].

## Conclusion

L’enquête ELVICA menée en 2007 dans la ville d’Antananarivo permet une meilleure connaissance de la violence conjugale dans la capitale de Madagascar. Elle montre que c’est un phénomène de grande ampleur (35% des femmes sont subies des violences conjugales au cours des 12 mois précédent l’enquête) et que c’est une vraie question de santé publique (19% des femmes ont été blessées par leur conjoint au cours des 12 derniers mois). Elle permet aussi d’identifier les femmes les plus à risque même si aucun groupe de femmes n’est totalement épargné. Ces informations ont des répercussions importantes en matière de prévention, de soins et de lutte contre la violence conjugale. L’ONG ENDA, partie prenante de cette enquête, s’en est d’ores et déjà saisie pour mettre en place des programmes d’information auprès de la population malgache, de formation des acteurs sociaux et de plaidoyer auprès des instances juridiques et policières.
